# Peanut panallergen Ara h 8 IgE and IgG4 epitopes

**DOI:** 10.1186/2045-7022-4-S2-P30

**Published:** 2014-03-17

**Authors:** Barry Hurlburt, Hsiaopo Cheng, Lesa Offermann, Maksymilian Chruszcz, Alexandra Santos, Gideon Lack, Soheila Maleki

**Affiliations:** 1 U.S. Department of Agriculture, Southern Regional Research Center, 1100 Robert E. Lee Blvd, New Orleans, 70124, USA; 2 U.S. Department of Agriculture, Food Allergy Research Unit, New Orleans, USA; 3 University of South Carolina, Chemistry and Biochemistry, Columbia, USA; 4 King's College, Paediatric Allergy, London, UK

## Background

Ara h 8 is hypothesized to be the panallergen responsible for oral allergy syndrome between birch pollen (Bet v 1) and peanut. We recently determined the crystal structure of Ara h 8. In this work, we probed microarrays of peptides with peanut allergic and peanut sensitized patient sera for IgE and IgG4 reactivity.

## Methods

15-mer peptides that were offset by 5 amino acids were printed to glass. Patient sera was incubated with the slides. IgE and IgG4 binding was detected with combinations of secondary and fluorescently-labeled tertiary antibodies. The linear epitopes identified were mapped on the 3-D structure and compared with those of birch pollen protein Bet v 1.

## Results

The majority of the Ara h 8 IgE epitopes mapped in this work align with those identified with Bet v 1. Considerably more IgG4 epitopes than IgE epitopes were found. Peanut allergic sera were more reactive with regard to IgE and IgG4 than peanut sensitized sera.

## Conclusion

Our results support both the hypothesis that Ara h 8 could be contributing to oral allergy syndrome between birch pollen and peanut.

